# Genomic Prediction Ability for Novel Profitability Traits Using Different Models in Nelore Cattle

**DOI:** 10.1111/jbg.70016

**Published:** 2025-09-22

**Authors:** Letícia Silva Pereira, Cláudio Ulhôa Magnabosco, Guilherme Rosa, Nedenia Bonvino Stafuzza, Tiago Zanett Albertini, Minos Carvalho, Raysildo Barbosa Lobo, Elisa Peripolli, Eduardo da Costa Eifert, Fernando Baldi

**Affiliations:** ^1^ Department of Animal Science Federal University of Goiás Goiânia GO Brazil; ^2^ Embrapa Cerrados Planaltina Brazil; ^3^ Department of Animal Science University of Wisconsin‐Madison Madison Wisconsin USA; ^4^ Department of Animal Science, Animal Science Institute (IZ), São Paulo's Agency for Agribusiness Technology (APTA) Sertãozinho São Paulo Brazil; ^5^ @Tech—Innovation Technology for Agriculture Piracicaba SP Brazil; ^6^ National Association of Breeders and Researchers (ANCP) Ribeirão Preto SP Brazil; ^7^ Department of Animal Science, São Paulo State University—Júlio de Mesquita Filho (UNESP), Access Way Prof. Paulo Donato Castelane Jaboticabal SP Brazil; ^8^ Department of Animal Science, Faculty of Animal Science and Food Engineering University of São Paulo Pirassununga São Paulo Brazil

**Keywords:** genomic selection, multi‐trait model, phenotypes of profit, prediction accuracy

## Abstract

The aim of this study was to assess the accuracy, bias and dispersion of genomic predictions for accumulated profitability (APF) and profit per kilogram of liveweight gain (PFT) in Nelore cattle using different prediction approaches. The dataset consisted of 3969 phenotypic records for each trait. The pedigree harboured information from 38,930 animals born between 1998 and 2016, including 2691 sires and 19,884 dams. A total of 2449 animals were genotyped using the Clarifide Nelore 3.0 SNP panel. Nine models for genomic prediction were evaluated: a linear animal model was applied to estimate genetic parameters and perform the genomic single‐trait best linear unbiased prediction (ST_ss—default). Additionally, a two‐trait (ssGBLUP TT_W450 and TT_DMI), three‐trait (TTT_CAR) and multi‐trait ssGBLUP (MT_ss) were tested. Finally, two models employing the weighted linear (ST_sswl1 and ST_sswl2) and non‐linear (ST_sswnl1 and ST_sswnl2) single‐step genomic approach (WssGBLUP) were used to predict genomic breeding values (GEBV). The ability to predict future performance was assessed by calculating the correlation between GEBV and adjusted phenotypes. The average prediction accuracy of the GEBV models ranged from 0.345 to 0.665 for PFT and from 0.425 to 0.603 for APF. The predictive capability of the MT_ss model (0.665) was significantly higher than that of the other models for PFT, except for the TTT_CAR model (0.604), which also showed an improvement in predictive performance. For APF, the MT_ss (0.561) and TT_W450 (0.556) models demonstrated improved genomic prediction accuracy compared to the other models. In general, the single trait ssGBLUP (ST_ss—default) models and the non‐linear weighting approach did not enhance prediction accuracy for either trait. For the phenotypic prediction ability of PFT, the linear WssGBLUP models ST_sswl1 (0.65) and ST_sswl2 (0.70), TT_W450 (0.64) and ssGBLUP‐M (0.66) demonstrated the highest prediction accuracies. Similar results were observed for the phenotypic prediction ability of APF for both models. However, the linear WssGBLUP models ST_sswl1 (0.84) and ST_sswl2 (0.94) provided higher prediction performance compared to the two‐, three‐ and multi‐trait models. The results indicate that the multi‐trait model achieved better predictive ability for the novel traits PFT and APF. Multi‐trait genomic selection may yield greater genetic gains than other models for these forthcoming economically important traits in breeding programmes.

## 1 Introduction

Beef cattle breeding is an indispensable component in the sustainability of the livestock industry worldwide (Mueller and Van Eenennaam 2022). Considerable advances in the tools and methods used for genetic improvement have contributed to the increased rate of genetic change in recent years (Van Eenennaam 2017). Additive genetic components were separated from environmental components using more sophisticated approaches, and objective measures replaced subjective ones (Van Eenennaam et al. 2014). Among these advances, genomics has emerged as the strategy with the greatest potential to accelerate genetic progress and productivity in livestock production (Weigel et al. 2010). It should be noted that genomic selection provides new opportunities as a starting point for selecting emerging traits, most of which have reference populations with a limited number of animals, due to the costs and challenges of practical phenotyping measurements in large volumes (Calus, Berry, et al. 2013; Calus, De Haas, et al. 2013), especially for complex traits with low heritability (Alvarenga et al. 2020).

Even 10 years after the introduction of genomics, the development of novel resources and their use is still ongoing (VanRaden 2020). Despite this progress, it is becoming increasingly challenging to meet both the market and livestock producers' needs, as well as consumer expectations (Merks et al. 2012). Livestock farming faces new challenges regarding sustainability, considering its three main components: social, environmental and economic (Boichard and Brochard 2012). In the current scenario, known as the ‘phenomics era’, access to more robust phenotypes has become necessary to address these expectations (Boichard et al. 2016). Therefore, new traits should be included as targets in selection objectives (Merks et al. 2012) with the help of precision or large‐scale tools (Seidel et al. 2020).

Given this, two proposed traits become strategic in addressing these issues, especially for the finishing phase, where feedlot operators face challenges such as management and input costs, carcass quality and market volatility. These traits are the accumulated profitability in the feedlot (AFP) and the profit per kilogram of live weight gain (PFT). Both traits are directly collected during the finishing phase in confined animals and are interpreted as indicators of an animal's genetic merit for profitability, based on performance and carcass traits, as well as economic data at both input and output stages of the finishing phase. In other words, these traits can be included to help in the early identification and selection of animals with high potential for economic and productive performance in the feedlot, while also contributing to financial and environmental sustainability.

Multiple methods have been suggested to estimate Genomic Estimated Breeding Values (GEBVs), such as Single‐Step Genomic Best Linear Unbiased Prediction (ssGBLUP) (Aguilar et al. 2010; Christensen and Lund 2010). The ssGBLUP model incorporates phenotype, pedigree and genomic information for individuals without genotypes but with phenotypic information, or individuals with only genotypic information through a pedigree‐based relationship matrix (*A*) with the genomic relationship matrix (*G*) in a hybrid matrix (*H*) (Legarra et al. 2009; Christensen and Lund 2010). However, this model assumes that the effects of single nucleotide polymorphisms (SNPs) have equal variance, which in biological terms may not be the most appropriate assumption (Meuwissen et al. 2001; VanRaden 2008; Goddard and Hayes 2009). Consequently, the weighted single‐step GBLUP (WssGBLUP) method proposed by Zhang et al. (2016), is an extension of ssGBLUP that assigns different weights to the SNPs used in calculating the G matrix.

Univariate analyses have been primarily used in genomic prediction models (Mehrban et al. 2021). However, this approach may not adequately capture the complex interactions between the analysed traits, as it does not account for the flow of information between them through the available information on genetic (co)variances (Gaire et al. 2022). (Co)variances arise from pleiotropy and linkage disequilibrium, resulting from the complex interactions of quantitative traits (Lynch and Walsh 1998). Therefore, the multi‐trait model has been shown to be more consistent in integrating information and identifying the effects of associations between traits, resulting in more accurate GEBV predictions than univariate analysis models (Calus and Veerkamp 2011; Guo et al. 2014; Wang et al. 2017). It is assumed that the multi‐trait approach could enhance the predictive capacity of GEBVs, especially for traits with low heritability or a limited number of records, through genetically correlated traits (Song et al. 2019).

Therefore, this study aimed to evaluate the predictive capacity of new traits related to profitability in feedlots in Nelore cattle. Nine models were evaluated, including best single‐step unbiased linear genomic prediction (ssGBLUP) and weighted single‐step linear and nonlinear genomic approaches (WssGBLUP) under one‐, two‐, three‐and multiple‐trait models. The analysis of these models focused on bias, dispersion and accuracy of genetic values to compare the profitability‐related traits.

## 2 Materials and Methods

This study was exempt from evaluation by the Animal Ethics Committee (CEUA), in accordance with Law No. 11,794 of 08/10/2008 and Normative Resolution No. 51 of 05/19/2021 from the National Council for Animal Experimentation Control (CONCEA) as all the analyses were conducted using pre‐existing databases.

### 2.1 Data Source

The dataset used in this study was provided by the National Association of Breeders and Researchers (ANCP, São Paulo, Brazil) in collaboration with @Tech (Innovation Technology for Agriculture LLC—Piracicaba, São Paulo, Brazil). The ANCP provided the pedigree information and genotypes, and novel feedlot profitability‐related phenotypes were provided by @Tech. For additional information about the company, please consult the official website: https://techagr.com/beeftrader. The animals belonged to 26 different herds located in the Southeast, Northeast and Midwest regions of Brazil. The pedigree contained information from 38,930 animals, born between 1998 and 2016, comprising 2691 sires and 19,884 dams.

### 2.2 Phenotyping

#### 2.2.1 Novel Traits

The new phenotypes analysed were accumulated feedlot profitability (AFP) and feedlot profit per kilogram of liveweight gain (PFT). AFP is the accumulated feedlot profitability in monetary units in the 80‐day period of feedlot and the PFT is the profitability per 15 kg of weight gained in feedlot.@Tech's algorithms are designed to make full use of this data and can be collected for up to 150 days, longer than the standard 80‐day data collection period in our study. The BeefTrader Decision Support System (Albertini et al. 2017) generates the profitability phenotypes used by the commercial tool Livestock Profit Tool (LPT) to identify the most profitable animals in the herd. The system uses animal growth modelling to define the best day for animals to leave the feedlot.

The BeefTrader algorithm uses animal traits as input variables (gender, breed, body condition score, age, initial weight, initial date, factors that impact on growth dynamics), daily weights individually collected through a weighing sensor (daily basis), and information on the nutritional composition of the diets (Albertini et al. 2017; Biase et al. 2022). The records of the dry matter intake for obtaining the new phenotypes were collected from animals participating in feed efficiency trials, following the same guidelines as Mendes et al. (2020), as mentioned in the section on feed efficiency traits. Based on this information, adjusted for local conditions, weight prediction was carried out in two steps: based on the biology of each animal and with the nutritional data and animal daily weight profile (observed or predicted), it is possible to estimate an optimal growth function for the animals (Step 1); from there, a dynamically adjusted linear or non‐linear regression is performed using the least squares method on the weights to fit the predicted growth curve (Step 2).

From the predicted growth curve, it is possible to find other variables required by the model, including animal performance in terms of growth and composition of gain, as well as economic and environmental factors (Gionbelli et al. 2016; Albertini et al. 2017). The equations for calculating phenotypes are presented below:

Accumulated arroba:

(1) $$\left(\mathrm{sbw}\times \mathrm{cdf}/100\right)/15$$

where sbw is the shrunk body weight (kg)—96% of the body weight. cdf is the carcass dressing (%).

The carcass dressing used was 55.34% for females and 58.55% for entire males. These are real values from the company's customer database and are close to those found in the literature, which range from 54.9% to 60.6% (Arcanjo et al. 2024; Anaruma et al. 2020; Rodrigues et al. 2003; Moreira et al. 2003).

To assess the profit obtained by meat producers, it is common to use the unit of measurement ‘arroba’ in Brazil. In this study, the ‘arroba’ unit is defined as equivalent to 15 kg, following the standard practice in the national livestock industry. Therefore, for the purposes of this study, the term ‘arroba’ in this equation will be used to represent the profit obtained per each 15 kg of meat produced.

Arroba×Gain:

(2) $$\text{accumulated kilograms}=\text{accumulated arroba}\;\left[d\right]-\text{initial gain}\;\left[1\right]$$

where accumulate arroba = accumulated $\text{kilograms}\;\left(\text{each}\;15\mathrm{kg}\right)$ on a specific day. [*d*] = final day of the period to be considered, in this case 80. [1] = first day of the feedlot.

Daily cost

(3) $$\mathrm{DMI}\times \text{diet price}\;\left(\mathrm{kg}\right)+\text{feedlot daily overhead}$$

where DMI = dry matter intake (kg), diet_price_kg = diet cost ($/kg), feedlot_daily_overhead = non‐feed cost ($).

Daily revenue:

(4) arrobaprice×arrobagain

where arroba_price = price of the arroba ($/15 kg), arroba gain = (referrig to each15kg/day).

Daily profit:

(5) revenue daily−cost daily

where revenue daily = daily revenue ($), cost daily = daily cost ($) and total revenue, cost and profit

(6) $$\sum_{\text{time}}\text{daily revenue}$$

(7) $$\sum_{\text{time}}\text{daily cost}$$

(8) $$\sum_{\text{time}}\text{daily profit}$$

where daily revenue = see Equation (4). Daily cost = see Equation (3). Daily profit = see Equation (5).

Cost and Profit per kilograms:

$$\text{total cost}/\text{arroba}\;{\text{gain}}_{t}$$

$$\text{total profit}/\text{arroba}\;\;{\text{gain}}_{t}$$

where total profit = see Equation (8). Total cost = see Equation (7).

${\text{arroba gain}}_{t}$= each15kg gain over time, see Equation (2).

#### 2.2.2 Standardisation of Costs and Arroba Pricing

##### 2.2.2.1 Food Cost

Even considering the effect of the batch (animals evaluated by farm) in the analyses, all common foods between batches, especially among farms, had their prices standardised to set up the food cost (e.g., for corn silage, the price was always the same for the different lots, and so for all common foods in the diet). Based on the cost of natural matter (as feed) and the percentage of dry matter (DM), from the measurement of each animal's daily individual intake, the food cost for everyone was imputed over the 80‐day evaluation period. It is important to note that after the adaptation period, there were 80 days of data collection on weight, DM intake (DMI), and food and non‐food costs (operational cost), all individual, to obtain the measure of accumulated profit and profitability per arroba gained by the evaluated animal.

##### 2.2.2.2 Non‐Food Cost (Operational Cost)

The non‐feed cost was also set at the same value for all evaluated batches with the aim of standardising this cost source in the process, and it is a source that doesn't affect the animals' performance.

##### 2.2.2.3 Price Paid per Kilograms (Arroba)

The arroba price for all batches was standardised to the prices at the time of data collection, with the aim of ensuring that the revenue per arroba was equal for all animals. The prices followed those indicated by the Center for Advanced Studies in Applied Economics (CEPEA, https://www.cepea.esalq.usp.br/br/indicador/boi‐gordo.aspx)—University of São Paulo (USP).

#### 2.2.3 Growth

A growth trait considered in this study was adjusted weight at 450 days of age (W450, kg). The standardised weight was calculated using linear regression, considering the average daily gain assessed between 405 and 495 days of age for the variable W450 (Negreiros et al. 2022).

#### 2.2.4 Carcass

The carcass traits considered in this study were ribeye area (REA, cm^2^) and rump fat thickness (RFT, mm). Carcass phenotypes were obtained through ultrasound images of the *Longissimus dorsi* muscle, taken between the 12th and 13th ribs (REA) and in the rump region, between the ilium and ischium at the intersection of the *Gluteus medius* and *Biceps femoris muscles* (RFT). These measurements were conducted using the ALOKA 500 V equipment with a 3.5 MHz linear probe.

#### 2.2.5 Feed Efficiency

The feed efficiency trait considered in this study was DMI. This trait was measured using the Intergado and GrowSafe electronic systems. Feed efficiency tests followed the guidelines established by Mendes et al. (2020) for assessing individual feed intake in beef cattle using both electronic systems. Animals were housed in either collective or individual pens and underwent to a 21‐day adaptation period, followed by a valid 70‐day testing phase. During this period, each animal's average weight was recorded either manually every 14 days or through automated weighing platforms (Intergado). Daily dry matter intake (DMI, kg/day) was calculated as the mean of all valid individual daily intake values electronically recorded by the Intergado and GrowSafe systems during the test period.

### 2.3 Statistical and Quality Control Analyses

The contemporary groups (CGs) were formed based on the effect of farm, year and season of birth (dry season: March to August, and rainy season: September to February), management group and sex. For the feed efficiency trait, the feed efficiency test identification was also considered in forming the CGs. Phenotypic quality control excluded records that deviated 3.5 standard deviations from the overall mean of the CGs and those with fewer than four records. Descriptive statistics for APF, PFT, carcass, growth and feed efficiency related traits used in the single, two‐, three‐ and multi‐trait analyses after quality control are summarised in Table 1.

**TABLE 1:** Descriptive statistics for profitability, growth, feed efficiency and carcass‐related traits in Nelore cattle.

TRAIT	*N*	MEAN	SD	MIN	MAX	CV (%)	CG
APF ($)	3969	157.71	67.18	−17.98	420.06	42.60	252
PFT ($/kg)	3969	36.65	9.54	−13.33	51.23	26.04	252
W450 (kg)	55,052	289.96	63.23	119.00	592.00	21.81	2211
DMI (kg/day)	11,169	8.17	2.06	3.18	20.66	25.23	251
REA (cm^2^)	37,091	57.55	12.85	20.45	114.97	22.33	1559
RFT (mm)	37,003	4.35	2.74	0.13	24.39	62.94	1559

Abbreviations: APF, accumulated profitability; BW450, weight at 450 days of age; CG, number contemporary group; CV, coefficient of variation; DMI, dry‐matter intake; MAX, maximum; MIN, minimum; *N*, number animals with records; PFT, profit per kilogram of liveweight gain; REA, rib eye area; RFT, rump fat thickness; SD, standard deviation.

### 2.4 Genotyping

Genotypes were provided by the National Association of Breeders and Researchers (ANCP), Ribeirão Preto, Brazil. A total of 2449 animals were genotyped using the low‐density panel (Clarifide Nelore 3.0). Genotype quality control (QC) was carried out by the PREGSF90 program (Aguilar et al. 2014), excluding both animals and SNPs from the dataset with call rates < 0.90. Additionally, SNPs with a minor allele frequency (MAF) < 0.05, Mendelian conflicts > 1%, monomorphic SNPs with redundant positions, SNPs deviating from Hardy–Weinberg equilibrium expectations (0.15), and those located on non‐autosomal chromosomes were also excluded. After QC, the dataset included 2449 genotyped animals and 35,658 SNPs for analysis.

### 2.5 Genomic Prediction Models

Genomic prediction models for APF and PFT were performed using nine genomic models applying the ssGBLUP methodology, which included a single‐trait model, two‐ and three‐trait models and a multi‐trait model. Additionally, in the case of the single‐trait model, both the weighted linear and non‐linear single‐step genomic approach (WssGBLUP) were applied in the analyses. The analyses were performed using the BLUPF90 family (Misztal et al. 2002), and the general model can be defined as:

(9) y=Xβ+Zu+e

where ***y*** is a vector of phenotypic records; ***β*** is a vector of fixed effects; ***X*** is a design matrix associating ***β*** with ***y**; **u*** is a vector of random effects of the direct additive genetic effects; ***Z*** is the incidence matrix associating ***u*** with ***y**; **e*** is the residual effect. Assumptions for residual effects are described below:

$$e\sim N\left(0,I\sigma_{e}^{2}\right),$$

where $\sigma_{e}^{2}$ is the residual variance, and ***I*** is an identity matrix with a dimension equal to the number of animals with records.

The description of the genomic prediction models is presented in Table 2.

**TABLE 2:** Genomic prediction models for feedlot profitability‐related traits in Nelore cattle.

Model	Description
*Single‐trait*	
ST_ss (default)	ssGBLUP based on genotypic records (default)
ST_sswl1	ssGBLUP weighting the diagonal of D matrix with the 𝜆 values obtained in the first iteration of the WssGWAS for nonlinear model
ST_sswl2	ssGBLUP weighting the diagonal of D matrix with the 𝜆 values obtained in the second iteration of the WssGWAS for linear model
ST_sswnl1	ssGBLUP weighting the diagonal of D matrix with the 𝜆 values obtained in the first iteration of the WssGWAS for nonlinear model
ST_sswnl2	ssGBLUP weighting the diagonal of D matrix with the 𝜆 values obtained in the 2st iteration of the WssGWAS for nonlinear model
Two‐trait	
TTT_CAR	
TT—W450	ssGBLUP based on genotypic records + BW450 records
TT_DMI	ssGBLUP based on genotypic records + DMI records
Three‐trait	
TTT_CAR	ssGBLUP based on genotypic records + carcass records
Mult‐trait	
MT_ss	ssGBLUP based on genotypic records + W450, DMI, REA and RFT records

#### 2.5.1 Single‐Trait Prediction Model

The ssGBLUP single‐trait model using information from both genotyped and non‐genotyped phenotype information and using both marker and pedigree information for genetic evaluations was performed. The ssGBLUP is a modification of the BLUP model, in which the inverse of the numerator relationship matrix *A* ^−1^ is replaced by *H* ^−1^ (Aguilar et al. 2010), which is given by:

$$H^{-1}=A^{-1}+\left[\begin{array}{cc} 0 & 0\\ 0 & G^{-1}-A_{22}^{-1} \end{array}\right]$$

where ***H*** is the relationship coefficient matrix between the animals; ***A*** is the (numerator) additive relationship matrix; $A_{22}^{-1}$ is a partition of *A* corresponding to the genotyped animals and $G^{-1}$ is the genomic relationship matrix described by VanRaden (2008) as *G* = ZZ′, where:

$$Z=\left(M-P\right)/{\left[2\;\sum_{j=1}^{K}p_{j}\left(1-p_{j}\right)\right]}^{1/2}$$

in which *M* is the matrix of *K* SNP genotype for each animal, and *P* is the matrix of frequency of the second allele *p* in the locus *j* (*p*_*j*_) multiplied by two.

#### 2.5.2 Two‐, Three‐ and Multi‐Trait Prediction Model

In the analysis of two‐, three‐ and multi‐trait models, carcass (REA and RFT), growth (W450) and feed efficiency (DMI) traits were identified as those genetically correlated with APF and PFT in the feedlot and were used as predictors in the genomic prediction models. The covariances and genetic correlations are presented in Table S1. Further details about the datasets used in this study are described in Pereira et al. (2025). Regarding the construction of the models, for the two‐trait analysis, the traits W450 and DMI were considered alongside APF and PFT in the feedlot, resulting in the following combinations: APF‐W450, APF‐DMI, PFT‐W450 and PFT‐DMI. The three‐trait analysis combined carcass (CAR) traits (CAR: REA and RFT) with APF and PFT, resulting in APF‐CAR and PFT‐CAR combinations. In the multi‐trait analysis, the traits W450, DMI, REA and RFT were considered in the APF and PFT models.

The two‐, three‐ and multi‐trait models were applied to estimate genomic breeding values for traits through the realised matrix (***H***) as follows (adapted from Guo et al. 2014):

$$\begin{array}{c} y_{1}\\ \vdots \\ y_{n} \end{array}=\left[\begin{array}{c} I_{1}\\ \vdots \\ 0 \end{array}\;\begin{array}{c} 0\\ \vdots \\ I_{N} \end{array}\right]\;\left[\begin{array}{c} \mu_{1}\\ \vdots \\ \mu_{n} \end{array}\right]+\left[\begin{array}{c} Z_{1}\\ \vdots \\ 0 \end{array}\;\begin{array}{c} 0\\ \vdots \\ Z_{n} \end{array}\right]\;\left[\begin{array}{c} g_{1}\\ \vdots \\ g_{n} \end{array}\right]\;+\left[\begin{array}{c} e_{1}\\ \vdots \\ e_{n} \end{array}\right]$$

where y is the vector that includes each of the *n* type traits. In these two‐, three and multi‐trait model, it was assumed that the genomic effects ~*N* (**0**, *G* ⊗ ***H***) and the residuals (*e*) ~ *N* (**0**, *R* ⊗ ***I***), where ⊗ is the Kronecker direct product; ***G*** and ***R*** are the genetic and residual covariance matrices, respectively; and ***I*** is an identity matrix. The assumed co‐variance structure is:

$$\mathrm{Var}\left[\begin{array}{c} g\\ e \end{array}\right]=\left[\begin{array}{cc} G⊗H & 0\\ 0 & {\mathrm{I\sigma}}_{e}^{2} \end{array}\right]$$

#### 2.5.3 Single‐Trait WssGBLUP Linear and Nonlinear

Before performing the weighted genomic prediction, a single‐step weighted GWAS (WssGWAS) was conducted to identify SNPs and their respective weights. For the ssGWAS, a single‐trait animal model was applied. The effects and variances of the SNPs were estimated following the methodology proposed by Wang et al. (2012). In this methodology, SNP effects are derived from the genomic values estimated by the ssGBLUP model, with SNP weights being iteratively updated. The iterative process increases the weights of SNPs with large effects and decreases those with smaller effects, effectivally regressing them towards the mean. Equation (9) was employed to construct the WssGBLUP model. For the derivation of SNPs effects and weights, the animal effect was decomposed into genotyped animals ($a_{g}$) and not genotyped ($a_{n}$), as described by Wang et al. (2012). The animal effect of the genotyped animals is a function of the SNP effects (Wang et al. 2012):

$$a_{g}=Z_{g}u$$

where $Z_{g}$ represents the relationship matrix of the genotypes of each locus, and ***u*** is a vector of the SNPs effects. The variance of animal effects was assumed as:

$$\mathrm{var}\;\left(a_{g}\right)=\mathrm{var}\left(Z_{g}u\right)=Z_{g}DZ_{g}\prime \sigma_{u}^{2}={G^{*}\sigma }_{a}^{2}$$

where *D* is a diagonal matrix of weights for variances of SNP variances (*D = I* for GBLUP), $\sigma_{u}^{2}$ is the variance of the additive genetic effect obtained from each SNP when the same variance is assumed for all SNPs, $\sigma_{a}^{2}$ is the additive genetic variance, and $G^{*}$ is the weighted genomic relationship matrix.

The ratio of covariance of additive genetic (a_***g***_) and SNPs (***u***) effects is:

$${\mathrm{var}}_{u}^{a_{g}}=\left[\begin{array}{cc} \mathrm{ZD}Z^{\prime } & ZD^{\prime }\\ DZ^{\prime } & D \end{array}\right]\sigma_{u}^{2}$$

Sequentially:

$$G^{*}=\frac{\mathrm{var}\left(a_{g}\right)}{\sigma_{a}^{2}}=\frac{\mathrm{var}\left(\mathrm{Zu}\right)}{\sigma_{a}^{2}}=\mathrm{ZD}Z^{\prime }\lambda$$

where *λ* is a normalising constant described by VanRaden et al. (2009) as:

$$\lambda =\frac{\sigma_{u}^{2}}{\sigma_{a}^{2}}=\frac{1}{\sum_{i=1}^{m}2p_{i}\left(1-p_{i}\right)}$$

where *m* is the number of SNPs and *p*_*i*_ is the frequency of the second allele in the *i*th SNP. The SNP effects can be described by Wang et al. (2012):

$$\hat{u}=\mathrm{\lambda D}Z^{\prime }G^{*-1}{\hat{a}}_{g}=DZ^{\prime }{\left[\mathrm{ZD}Z^{\prime }\right]}^{-1}{\hat{a}}_{g}$$

The estimated SNP effects can be used to calculate the variance of each individual SNP (Zhang et al. 2010), which can be used as different weighting for each SNP:

$$\sigma_{u,i}^{2}={\hat{U}}_{i}^{2}2p_{i}\left(1-p_{i}\right)$$

where $\sigma_{u,i}^{2}$ is the *j* SNP weight (equivalent to *j* SNP variance); û is a vector of estimated *j* SNP effect; and *p* is the allele frequency of *j* SNP.

Two strategies were used to weight SNPs and perform genomic prediction: linear and non‐linear methodology. Weighted ssGWAS is an iterative process with several steps, considering *t* as the iteration number; the steps are (Wang et al. 2012):

1. Let D = I in the first step.
2. Calculate G = $Z_{g}DZ_{g}\prime \lambda$.
3. Calculate GEBVs for the entire data set using the ssGBLUP.
4. Convert GEBVs to SNP effects (*û*): *û* = λ *DZ*′ ($Z_{g}$ *D* $Z_{g}$′ λ) ^−1^ ${â}_{g}$, where ${â}_{g}$ is the GEBVs of animals which were also genotyped.
5. Calculate weight for each SNP for linear model (Zhang et al. 2010): $d_{i}={\hat{a}}_{i}^{2}2p_{i}\left(1-p_{i}\right)$ (default)

For the non‐linear weighted model, using similar approach to VanRaden (2008), SNP‐specific weights were calculated as:

$$d_{i}=1.125^{\frac{|{\hat{a}}_{i}|}{\mathrm{sd}\;(\hat{a)}}-2}$$

6 Normalise SNP weights to maintain the total genetic variance constant.
7 Back to step 2.

The analysis involved two iterations of the WssGBLUP model. The first iteration used an identity matrix to weight SNP effects, and the second used the D matrix derived from the SNP solutions estimated in the first step. This second iteration led to higher GEBV accuracies in the preliminary analysis. The SNP solutions were estimated using the POSTGSF90 software (Aguilar et al. 2014), and the genomic association analyses were performed using the BLUPF90 software family (Misztal et al. 2002), incorporating the genomic information as outlined by Aguilar et al. (2010). The results were presented as the proportion of the additive genetic variance explained by windows of 10 adjacent SNPs, as below:

$$\frac{\mathrm{Var}\left(a_{i}\right)}{\sigma_{a}^{2}}\times 100=\frac{\mathrm{Var}\left(\sum_{j=1}^{10}Z_{g}c\right)}{\sigma_{a}^{2}}\times 100$$

where $a_{i}$ is the genetic value of the *i*th region that consists of continuous 10 SNPs; $\sigma_{a}^{2}$ is the additive genetic variance; and c is the marker effect of the *i*th SNP within the *i*th region.

Manhattan plots of the first and second iterations of linear and nonlinear WssGWAS, along with the respective percentages of additive genetic variance explained by the 10‐SNP windows for APF and PFT are presented in Supporting Information.

### 2.6 Prediction Accuracy, Bias and Dispersion

To conduct the prediction analyses, the dataset was divided into whole (*w*, training) and partial (*p*, validation) subsets. The validation subset was created by removing the phenotypic records of the target validation animals from the dataset, ensuring that evaluations were initially performed with the partial dataset. In this process, the partial GEBV (GEBV*p*) is calculated based on relatives and genomic information. Subsequently, the phenotypes of the validation animals are included, and the whole genomic breeding value (GEBV*w*) is achieved. Animals born in 2022 and 2023 from six herds were selected as the target validation group, representing young selection candidates. This validation methodology simulates the practical scenario in genomic selection, where the prediction of GEBV for younger animals (i.e., selection candidates) is based on phenotypic and genotypic information from proven or older animals within the total population. Table 3 presents the descriptive statistics of the traits for both the whole and partial datasets.

**TABLE 3:** Number of animals with phenotypic records in the whole and partial subsets for profitability‐related traits in Nelore cattle.

Dataset	Trait	*N*	Mean	SD	Min	Max	CV (%)	CG
Whole subset	APF	3478	160.26	62.54	−17.98	420.06	39.02	242
	PFT	3478	37.62	8.83	−13.34	51.23	23.47	242
Partial subset	APF	502	139.64	91.75	−6.59	374.61	65.70	28
	PFT	502	29.76	11.39	−3.49	48.57	38.28	28

Abbreviations: APF, accumulated profitability; CG, number contemporary group; CV, coefficient of variation; MAX, maximum; MIN, minimum; *N*, number animals with records; PFT, Profit per kilogram of liveweight gain; SD, standard deviation.

Prediction accuracy, bias and dispersion were calculated according to the methodology proposed by Legarra and Reverter (2018). Prediction accuracy (${\mathrm{acc}}_{p}^{2}$) represents the squared population accuracy of the GEBV estimated from a partial dataset. This method uses the covariance between GEBVs obtained from partial and whole datasets (${\rho_{\mathrm{Cov}}^{2}}_{w,p}$) which is modelled as a function of the reliability (squared accuracy) of the partial‐data GEBV, as shown below::

$${\mathrm{acc}}_{p}^{2}={\rho_{\mathrm{Cov}}^{2}}_{w,p}=\sqrt{\frac{\mathrm{cov}\left({û}_{w},{û}_{p}\right)}{\left(1-\bar{F}\right){\sigma_{a}}^{2}}}$$

where û_*w*_ is the GEBV estimated using the whole dataset with genomic and phenotypic information; *ûp* is the (G)EBV considering the partial dataset; *F* is the average inbreeding coefficient estimated for the animals included in the validation dataset; and ${\sigma_{u}}^{2}$ is the additive genetic variance for APF and PFT.

The mean of the estimated breeding values was used to evaluate bias, which has an expected value of 0 under an unbiased evaluation:

$$\mu_{\mathrm{wp}}={\bar{\hat{u}}}_{p}-{\bar{\hat{u}}}_{w}$$

The dispersion was calculated by regressing the EBVs from whole dataset on EBVs from the partial dataset. The $b_{w,p}$ has an expected value $E\left(b_{w,p}\right)=1$, assuming there is no over/under dispersion:

$$b_{w,p}=\frac{\mathrm{cov}\left({û}_{w},{û}_{p}\right)}{\mathrm{var}\left({û}_{p}\right)}$$

To assess the predictive capacity of the phenotypes across the different models, Pearson's correlation between the GEBV and the adjusted phenotype for the fixed effects (Yc) was used (Legarra et al. 2008). Phenotypic adjustment was carried out using the PREDICTF90 software from the BLUPF90 suite (Misztal et al. 2002). The correlation between the GEBV and the adjusted phenotype (y*, defined as the phenotype y corrected for fixed effects) was calculated for individuals in the validation population. This correlation was subsequently divided by the square root of the heritability (*h* ^2^) ($h^{2}$):

$${\mathrm{ACC}}_{P}=\frac{r\left({\hat{u}}_{p},y^{*}\right)}{\sqrt{h^{2}}}$$

## 3 Results

The prediction accuracy, bias and dispersion of genomic prediction for PFT and APF using different prediction models are described in Table 4. The results revealed that the prediction accuracy of the ST_sswl1 (0.587) and ST_sswl2 (0.528) models was higher than that of the ST_ss model (0.345) for PFT. The WssGBLUP model provided an expressive increase of 59% in prediction accuracy, as shown in Table 4. A similar trend was observed for APF, whose prediction accuracy of the ST_sswl1 (0.575) and ST_sswl2 (0.603) models was higher than the single‐trait model ssGBLUP (0.425). On the other hand, in terms of prediction accuracy, the nonlinear models ST_sswnl1 (0.365 and 0.443, respectively) and ST_sswnl2 (0.365 and 0.442, respectively) showed no improvements compared to the ssGBLUP model for both traits. When incorporating information from correlated traits in the two‐, three‐ and multi‐trait models, such as W450 and carcass with PFT, improvements in the accuracy of the GEBVs were observed, reaching 0.604 (TTT_CAR) and 0.523 (TT_W450). Similarly, gains were observed in the predictive capacity for APF with W450, DMI and carcass, with accuracy values of 0.503 (TT_DMI), 0.505 (TTT_CAR) and 0.556 (TT_W450). As shown in the genetic correlations in Table S1, the genomic prediction for PFT indicated that the two‐trait model (TT_DMI) did not significantly improve prediction accuracy, possibly due to low genetic correlations. This may be due to the low genetic association between these traits and the limited amount of DMI data, resulting in lower accuracy of 0.372.

**TABLE 4:** Accuracy, bias and dispersion of genomic predictions for feedlot accumulated profitability and profit per kilogram of liveweight gain in Nelore cattle across different models.

Trait	Model	Accuracy	Bias	Dispersion
Profit per kilogram of liveweight gain	*Single‐trait*			
	*ST_ss*	0.345 (0.000)	−0.771 (0.250)	1.124 (0.043)
	*ST_sswl1*	0.587 (0.000)	−0.792 (0.242)	0.713 (0.013)
	*ST_sswl2*	0.528 (0.000)	−0.746 (0.234)	0.470 (0.014)
	*ST_sswnl1*	0.365 (0.000)	−0.770 (0.249)	1.111 (0.038)
	*ST_sswnl2*	0.365 (0.000)	−0.770 (0.249)	1.120 (0.038)
	*Two‐trait*			
	*TTT_CAR*			
	*TT_W450*	0.523 (0.000)	−1.031 (0.349)	1.063 (0.019)
	*TT_DMI*	0.372 (0.000)	−1.075 (0.346)	1.122 (0.034)
	*Three‐trait*			
	*TTT_CAR*	0.604 (0.005)	−1.230 (0.345)	0.969 (0.019)
	*Mult‐trait*			
	*MT_ss*	0.665 (0.00)	−1.111 (0.344)	0.982 (0.016)
Accumulated profitability	*Single‐trait*			
	*ST_ss*	0.425 (0.000)	0.002 (0.018)	1.104 (0.045)
	*ST_sswl1*	0.575 (0.000)	0.049 (0.017)	0.739 (0.023)
	*ST_sswl2*	0.603 (0.000)	0.033 (0.024)	0.556 (0.022)
	*ST_sswnl1*	0.443 (0.000)	0.001 (0.017)	1.084 (0.040)
	*ST_sswnl2*	0.442 (0.000)	0.001 (0.017)	1.093 (0.040)
	*Two‐trait*			
	*TTT_CAR*			
	*TT_W450*	0.556 (0.000)	0.002 (0.015)	1.087 (0.028)
	*TT_DMI*	0.503 (0.000)	−0.003 (0.016)	1.065 (0.034)
	*Three‐trait*			
	*TTT_CAR*	0.505 (0.000)	0.096 (0.015)	1.068 (0.032)
	*Mult‐trait*			
	*MT_ss*	0.5612 (0.000)	0.004 (0.015)	1.049 (0.027)

Abbreviations: *λ* = proportion of additive genetic variance explained by each SNP. TTT_CAR. MT_ss = ssGBLUP with genotypic, BW450, DMI, REA and RFT records; ST_ss = ssGBLUP (default, based on genotypic records); ST_ssw1 = ssGBLUP weighting the diagonal of the D matrix with *λ* values from the first iteration of WssGWAS (nonlinear); ST_ssw2 = ssGBLUP with *λ* from the second iteration (nonlinear); TT_DMI = ssGBLUP with genotypic + DMI records; TT_W450 = ssGBLUP with genotypic + BW450 records; TTT_CAR = ssGBLUP with genotypic + carcass records.

The dispersion values varied between PFT and APF within the models, as shown in Table 4. These values suggest variation in predictive ability, with both inflation and deflation of the estimated GEBVs. Values above one indicate underdispersion (deflation), while values below one indicate overdispersion (inflation) of the estimated genetic values Himmelbauer et al. (2023). For both traits, the ST_ss, ST_sswnl1, ST_sswnl2, TTT_CAR, TT_W450 and TT_DMI provided relatively larger slopes for predictions, with values ranging from 1.063 to 1.124 for PFT and from 1.065 to 1.104 for AFP, respectively. The ST_sswl1 and ST_sswl2 obtained predictions with the regression coefficients furthest from one, with values of 0.713 and 0.470 for PFT and 0.556 and 0.739 for APFT, respectively. The MT_ss models were generally less dispersed (values closer to 1), with values of 0.982 (PFT) and 1.049 (APF).

The prediction accuracies for the fraction of additive genetic variance explaining the phenotype were obtained by dividing the correlation between the partial GEBV and the phenotype adjusted for fixed effects by the square root of the heritability. The ST_sswl2 presented the highest precision values of the fraction of additive genetic variance that explains the adjusted phenotype for PFT, with a value of 0.70, followed by 0.66 (MT_ss), 0.65 (ST_sswl) and 0.64 (TT_W450). Slightly lower predictive ability was pbserved for the ST_sswnl1 (0.58) and ST_sswnl2 (0.55) models, while the lowest predictive ability values were observed for the TTT_CAR (0.41), TT_DMI (0.38) and ST_ss (0.33) models. For APF, weighted linear models followed the same trend, with the ST_sswl (0.84) and ST_sswl2 (0.94) models showing higher predictive accuracies compared to TT_W450 (0.57), MT_ss (0.56), ST_sswnl1 (0.54), ST_sswnl2 (0.54), TT_DMI (0.52), TTT_CAR (0.46) and ST_ss (0.46) modelsFor this dataset, the weighted linear model WssGBLUP was more efficient in predicting the fraction of additive genetic variance responsible for explaining phenotype expression.

## 4 Discussion

The prediction values and accuracy obtained under the different models support the idea that a model's performance depends on its ability to capture and consider the genetic structure of the traits under study, including the effects of polymorphisms, regardless of their magnitude, whether those with large or small effect (Terakado et al. 2021). Based on the observed results, it can be inferred that the relevant increase in the prediction accuracy of the linear models ST_sswl1 and ST_sswl2 compared to the nonlinear model can be attributed to the polygenic nature of the traits under study, in which the additive genetic variation for traits related to feedlot profitability in Nelore cattle is largely explained by numerous small‐effect SNPs.

The differences observed in the predictive capacity of GEBVs from the two‐ and three‐trait models can be explained by the traits with the strongest genetic correlation with PFT, such as W450 and carcass‐related traits, as well as APF with DMI, W450 and carcass‐related traits. Additionally, the number of records contributed by each trait in the models may also have influenced these differences.

Despite the moderate genetic correlation between PFT and carcass‐related traits, as well as the lower number of records compared to W450, significant improvements in predictive capacity were observed when carcass traits (TTT_CAR) were included in the model. Regarding APF, despite the high genetic correlation with W450 (Table S1), the TTT_CAR and TT_DMI models also contributed to its prediction accuracy. The highest prediction accuracy for both traits was obtained in the MT_ss model, demonstrating superior predictive ability over the single‐trait linear model WssGBLUP and the two‐trait model ssGBLUP.

The results presented herein align with those reported by Guo et al. (2014) and Song et al. (2019), in which the two‐, three‐ and multi‐trait genomic models proved more effective for traits with a smaller set of phenotypic data. This approach is suitable for genetic improvement programs, as in a reference population, most individuals will often not have phenotypes available for all traits of interest (Guo et al. 2014). Moreover, it is particularly relevant for research on novel traits to be incorporated as selection criteria, considering their costs and the difficulty of measurement due to the varying environments and regions in which different production systems operate. According to Guo et al. (2014), incorporating records of genetically correlated and easily measured traits, as well as multi‐trait models, contributes to improvements in the prediction ability of GEBVs. However, the differences between the multi‐trait and single‐trait models are not likely to significantly differ in terms of prediction accuracy for traits with complete records and high heritability.

Regarding bias, it was observed to be low and followed the same trends for all prediction models of the PFT, except for APF, whose values were close to zero for all models, ranging from −0.003 to 0.096. Different variables may limit the response of prediction accuracy and reduce the bias of genomic predictions, as reported by Lund et al. (2009), Zhang et al. (2011) and Goddard (2009), who noted that the genetic architecture of the traits of interest can be characterised by heritability and the number of associated QTLs. This finding is consistent with the biases observed in our results, which may be attributed to the low genetic variance and heritability of the PFT trait. TTT_CAR. This result suggests a slight tendency towards inflation and more significant variability in predictions.

However, it is important to note that, although these models provided greater predictive ability compared to the others, the dispersion values exhibited greater underdispersion, suggesting probable deflation in the estimated genetic values. When predictions are deflated and greater than one, in practical terms, the difference between the progeny of selected sires is expected to be greater than what is predicted by the GEBV. Conversely, the opposite occurs when the predictor is inflated (Chiaia et al. 2017). Although the predictive capacity values are similar between the multi‐trait and two‐ and three‐trait models, the regression coefficient values close to one for the model suggest the better consistency of this model in predicting GEBVs. Therefore, the two‐trait model was more effective in predicting the absolute differences between individuals under evaluation.

These results also indicate that incorporating information from weighted regions of GWAS into genomic prediction models through the weighted linear WssGBLUP approach can be an effective strategy to improve the accuracy of predicting the fraction of additive genetic variance for the traits under study. Van den Berg et al. (2016), Raymond et al. (2018) and Peripolli et al. (2024) reported that sequence variants selected from a GWAS could be used to improve predictive capacity, as well as to predict the fraction of additive genetic variance responsible for explaining the phenotype.

The genetic architecture of a complex trait exerts a considerable influence on genomic prediction (Hayes et al. 2010). Potential genomic regions harbouring genes with informative SNPs for APF and PFT were identified, as illustrated in the Manhattan plots in the supplemental material (Figures S1–S8), with potential candidate genes involved in biological processes such as carbohydrate, protein, and lipid metabolism, immunity and feeding behaviour. Additionally, these genomic regions may be associated with traits related to growth, reproduction, feed efficiency and carcass.

These results suggest that the analyses of two‐, three‐ and multi‐trait and nonlinear weighted models did not contribute to the predictive accuracies of the adjusted phenotypes. On the other hand, weighted regions, where weighted linear models used their own QTL information, contributed to a better predictive capacity of the fraction of additive genetic variance explaining the adjusted phenotypes for PFT and APF. This indicates that these models were more efficient in capturing the additive genetic variation that effectively contributes to the phenotypic expression of each trait.

Regarding the models evaluated, although multitrait models displayed better predictive capacity, their use requires the estimation of a large number of (co)variance components. This implies higher computational demands, such as processing time and memory usage, due to the need to estimate all variance and covariance matrices among all the traits under study. According to Meyer (2007), multivariate analyses have been limited by high computational requirements coupled with the difficulty in accurately estimating a large number of covariance components simultaneously. Alternatively, principal component analysis (PCA) has been proposed as a dimensionality reduction strategy, aiming to simplify model complexity (Meyer 2007). It is considered an alternative to reduce both the number of estimated EPDs and the number of parameters required to model the genetic covariance matrix (Kirkpatrick and Meyer 2004).

Similarly, models that use genomic weighting (WssGBLUP) may be related to hyperparameterisation, as these models assume that certain genomic regions contribute unequally to the total genetic variance, which may have contributed to greater bias and dispersion. Furthermore, the weighted model requires steps prior to estimating genomic predictions, such as analysing the weighting of genomic regions, since the weighted genomic matrix increases the complexity of the process and, consequently, requires higher computational demand.

From a practical standpoint, these factors may impair the application of these multi‐trait models in large‐scale commercial selection programmes, especially for those with more limited computational infrastructure or less availability of phenotypic data and genetically correlated traits. Despite the limitations associated with multi‐trait models, it is essential to consider that incorporating information from genetically correlated traits can significantly enhance the predictive accuracy of genomic prediction for novel phenotypes with smaller databases or traits that are difficult to measure. Therefore, genetic improvement programmes should carefully consider the balance between gains in predictive capacity and the technical and operational feasibility of implementing these models in commercial settings.

## 5 Conclusions

The results obtained herein showed that multi‐trait models improve the genomic prediction capacity for profitability‐related traits in feedlots. Genomic prediction using multi‐trait models is beneficial when the trait is complex and the number of phenotypes (i.e., novel traits) is limited. This strategy is more accurate for improving genomic prediction estimates of these traits and integrating them efficiently into genetic improvement programmes. These results provide additional support to breeders in improving management and selection decisions to enhance feedlot profitability operations. The incorporation of weighted regions for GEBV prediction in the single‐trait weighted linear model helps to improve predictive capacity; however, more biased predictions and dispersion values with more significant inflation were observed.

From a practical point of view, for commercial herds, feedlots, and calf comparators for rearing and finishing, the weighted single‐trait models (ST_sswnl2) were superior in predicting the phenotype. The results obtained for predicting future performance (phenotype) can help producers make earlier and more assertive management decisions, improving production efficiency and economic return per animal in their operations, by prioritising animals with higher genetic merit for profitability‐related traits in feedlots.

## Author Contributions

Letícia Silva Pereira: conceptualisation, methodology, formal analysis, writing‐original draft preparation. Cláudio Ulhôa Magnabosco: conceptualisation, methodology, formal analysis, writing and review. Guilherme Rosa: methodology, formal analysis, writing and review. Nedenia Bonvino Stafuzza: conceptualisation, writing and review. Tiago Zanett Albertini: data curation, writing‐review and editing. Minos Carvalho: data curation, writing‐review and editing. Raysildo Barbosa Lobo: data curation, review. Elisa Peripolli: conceptualisation. Eduardo da Costa Eifert: conceptualisation, writing and review. Fernando Baldi: conceptualisation, methodology, formal analysis, writing‐original draft preparation.

## Conflicts of Interest

The authors declare no conflicts of interest.

## Supporting information


**Data S1:** jbg70016‐aup‐0001‐Supinfo.docx.

## Data Availability

The data that support the findings of this study are available on request from the corresponding author. The data are not publicly available due to privacy or ethical restrictions.
