# Supplementary material for: Genomic Prediction Ability for Novel Profitability Traits Using Different Models in Nelore Cattle
Source: J Anim Breed Genet. 2025 Sep 22;143(2):244–55. doi: 10.1111/jbg.70016 (PMC12887148; doi:10.1111/jbg.70016)
Supplement: Supplementary file 1 — Data S1: jbg70016‐aup‐0001‐Supinfo.docx. [file JBG-143-244-s001.docx]

**Supplementary material**

Table S1. Covariance components and genetic and residual correlations between accumulated profitability (APF), profit per kilogram of liveweight gain (PFT) and e adjusted weight at 450 days of age (W450), dry matter intake (DMI), ribeye area (REA) and rump fat thickness (RFT).

| **Trait** | **Components** | **W450** | **DMI** | **REA** | **RFT** |
| --- | --- | --- | --- | --- | --- |
| PFT | ${Cov}_{a}$ | 35.78 | 0.47 | 5.13 | -1.19 |
|  | ${Cov}_{e}$ | 43.99 | 0.92 | 5.17 | 1.49 |
|  | $r_{a}$ | 0.64 ±0.05 | 0.26 ±0.08 | 0.44 ±0.07 | -0.68 ±0.04 |
|  | $r_{e}$ | 0.09 ±0.03 | 0.06 ±0.02 | 0.05 ±0.02 | 0.07 ±0.03 |
| APF | ${Cov}_{a}$ | 882.94 | 27.19 | 115.90 | -5.74 |
|  | ${Cov}_{e}$ | 827.79 | 27.52 | 101.63 | 22.62 |
|  | $r_{a}$ | 0.51 ±0.82 | 0.72 ±0.09 | 0.43 ±0.09 | -0.10 ±0.07 |
|  | $r_{e}$ | 0.21 ±0.03 | 0.21 ±0.03 | 0.12 ±0.03 | 0.13 ±0.03 |

${Cov}_{a}$, additive genetic covariance; ${Cov}_{e}$, residual covariance; $r_{a}$ additive genetic correlation; $r_{e}$ , residual correlation.

Figure S1 Manhattan plot of the genomic regions of 10 adjacent SNPs that explain more than 0.5% of the additive genetic variance (Var) obtained in the 1^nd^ iteration of the linear WssGWAS for accumulated profitability in Nelore cattle.


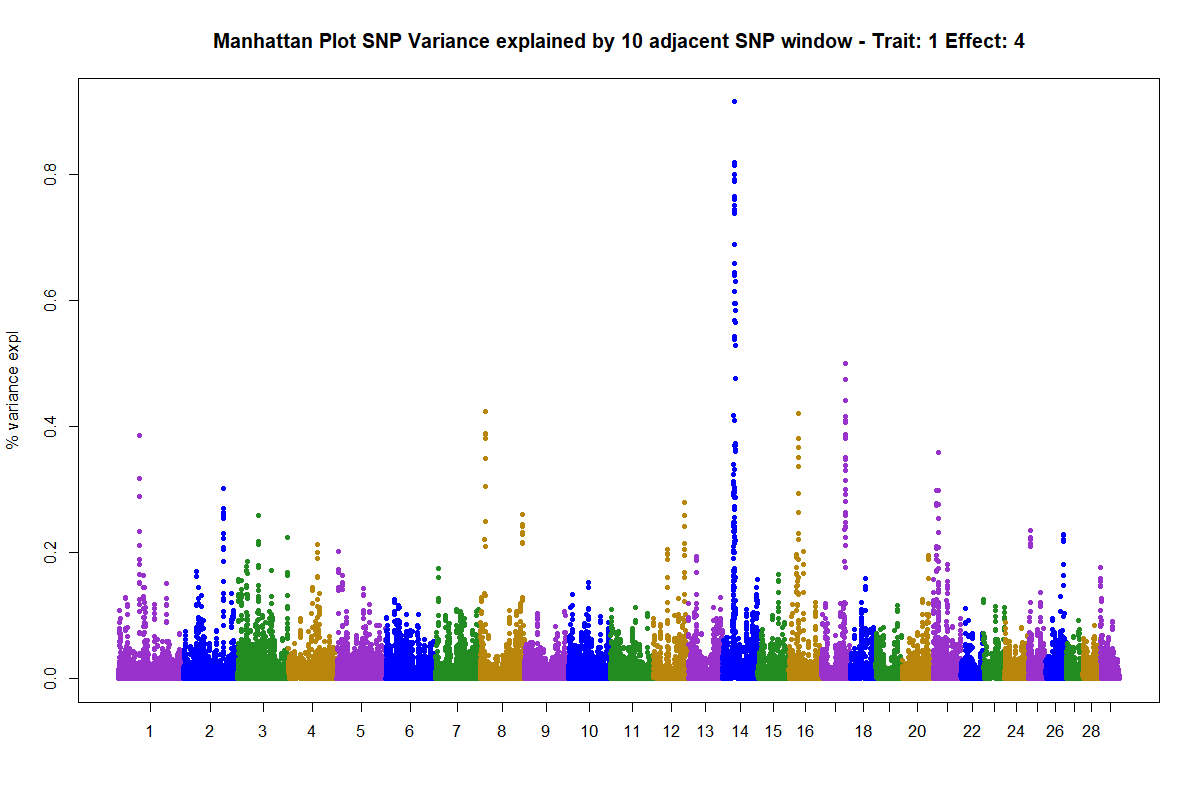


Figure S2. Manhattan plot of the genomic regions of 10 adjacent SNPs that explain more than 0.5% of the additive genetic variance (Var) obtained in the 2^nd^ iteration of the linear WssGWAS for accumulated profitability in Nelore cattle.


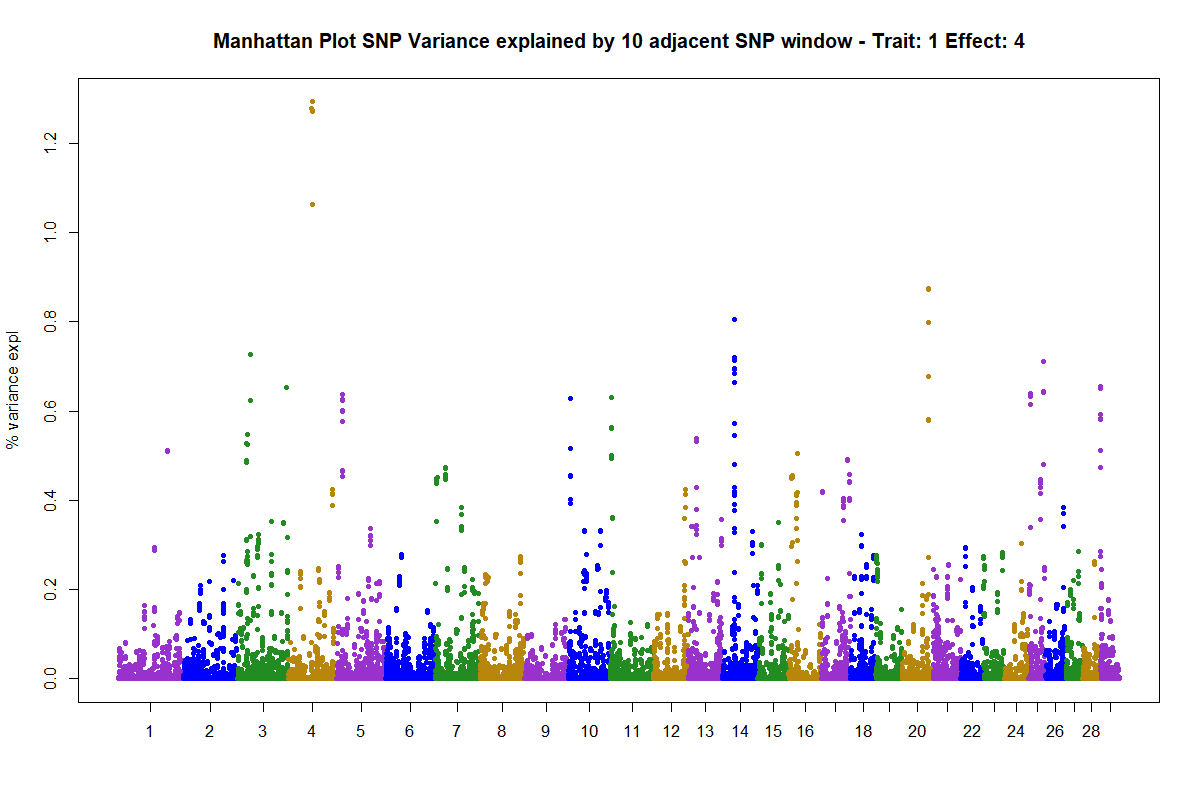


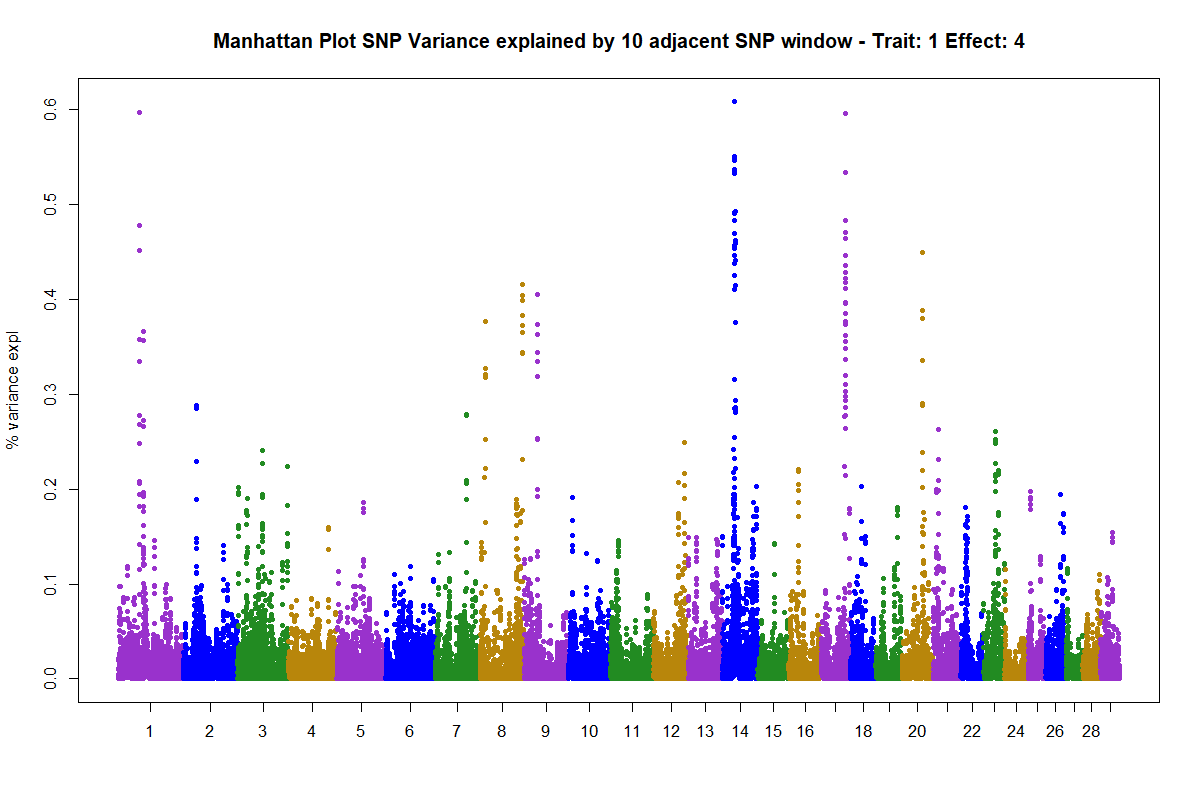
Figure S3. Manhattan plot of the genomic regions of 10 adjacent SNPs that explain more than 0.5% of the additive genetic variance (Var) obtained in the 1^nd^ iteration of the linear WssGWAS for profit per kilogram of liveweight gain in Nelore cattle.


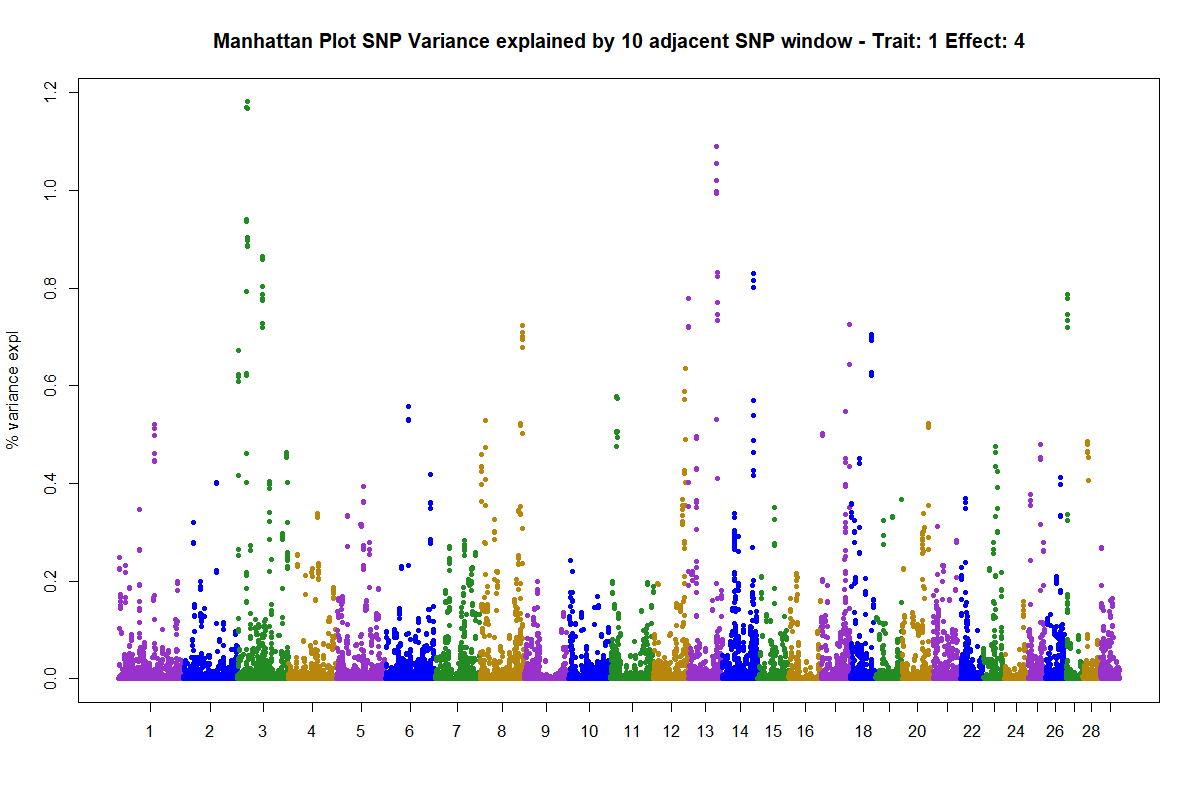


Figure S4. Manhattan plot of the genomic regions of 10 adjacent SNPs that explain more than 0.5% of the additive genetic variance (Var) obtained in the 2^nd^ iteration of the linear WssGWAS for profit per kilogram of liveweight gain in Nelore cattle.


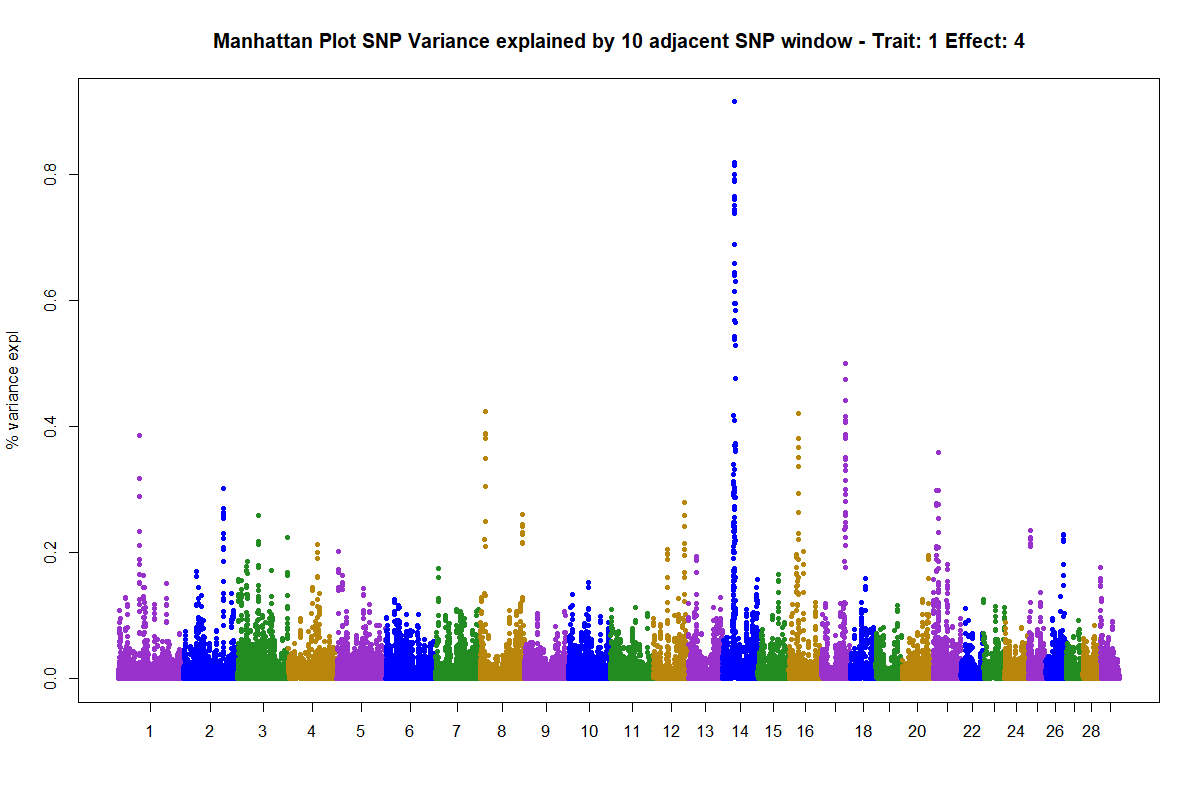


Figure S5. Manhattan plot of the genomic regions of 10 adjacent SNPs that explain more than 0.5% of the additive genetic variance (Var) obtained in the 1^nd^ iteration of the nonlinear WssGWAS for accumulated profitability in Nelore cattle.


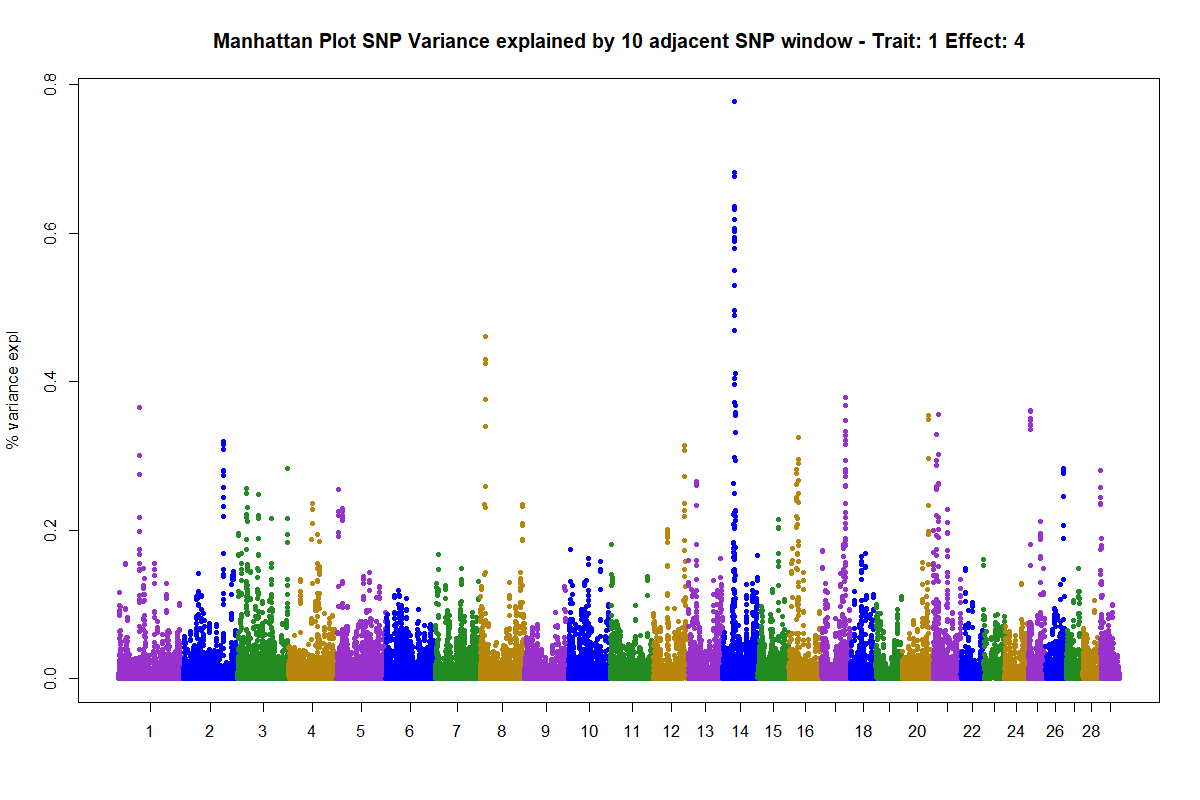


Figure S6. Manhattan plot of the genomic regions of 10 adjacent SNPs that explain more than 0.5% of the additive genetic variance (Var) obtained in the 2^nd^ iteration of the nonlinear WssGWAS for accumulated profitability in Nelore cattle.


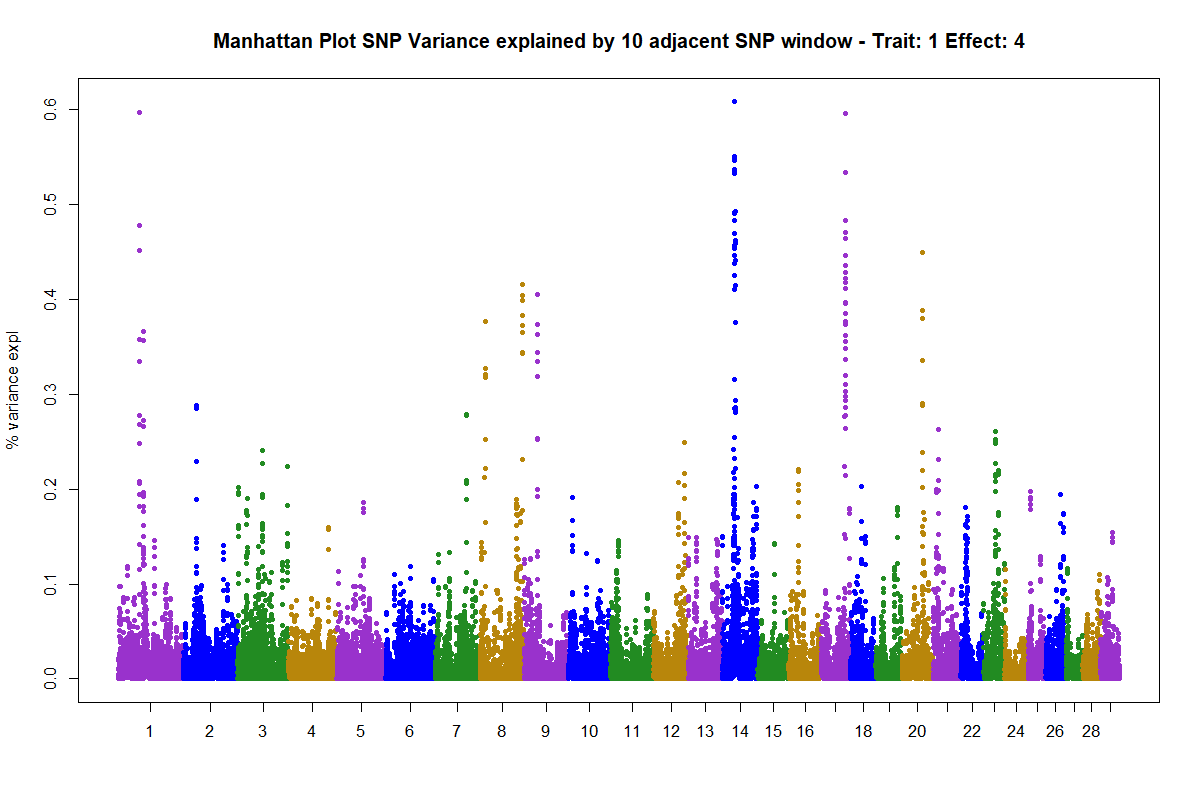


Figure S7. Manhattan plot of the genomic regions of 10 adjacent SNPs that explain more than 0.5% of the additive genetic variance (Var) obtained in the 1^nd^ iteration of the nonlinear WssGWAS for profit per kilogram of liveweight gain in Nelore cattle.


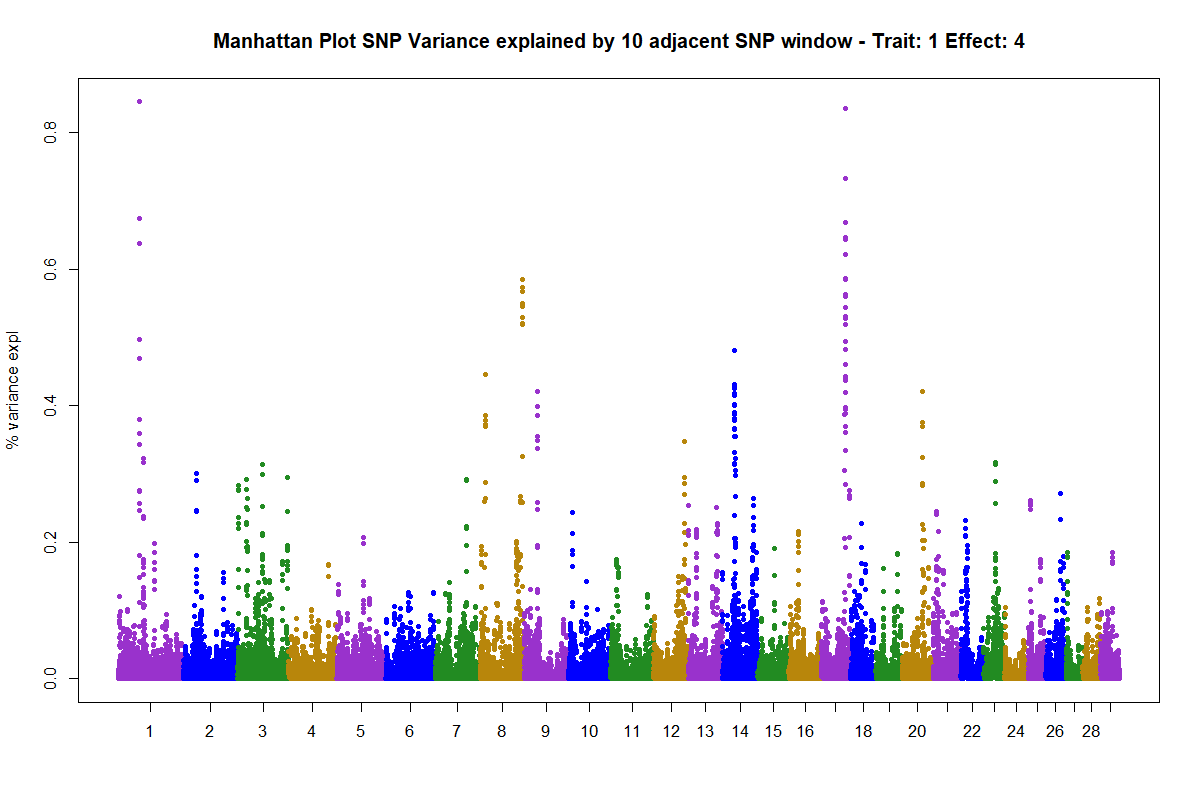


Figure S8. Manhattan plot of the genomic regions of 10 adjacent SNPs that explain more than 0.5% of the additive genetic variance (Var) obtained in the 2^nd^ iteration of the nonlinear WssGWAS for profit per kilogram of liveweight gain in Nelore cattle.
